# Be Happy Not Sad for Your Youth: The Effect of Emotional Expression on Age Perception

**DOI:** 10.1371/journal.pone.0152093

**Published:** 2016-03-30

**Authors:** Norah C. Hass, Trent D. Weston, Seung-Lark Lim

**Affiliations:** Department of Psychology, University of Missouri-Kansas City, Kansas City, Missouri, United States of America; University of Pécs Medical School, HUNGARY

## Abstract

Perceived age is a psychosocial factor that can influence both with whom and how we choose to interact socially. Though intuition tells us that a smile makes us look younger, surprisingly little empirical evidence exists to explain how age-irrelevant emotional expressions bias the subjective decision threshold for age. We examined the role that emotional expression plays in the process of judging one’s age from a face. College-aged participants were asked to sort the emotional and neutral expressions of male facial stimuli that had been morphed across eight age levels into categories of either “young” or “old.” Our results indicated that faces at the lower age levels were more likely to be categorized as old when they showed a sad facial expression compared to neutral expressions. Mirroring that, happy faces were more often judged as young at higher age levels than neutral faces. Our findings suggest that emotion interacts with age perception such that happy expression increases the threshold for an old decision, while sad expression decreases the threshold for an old decision in a young adult sample.

## Introduction

Perceived age is a psychosocial factor that carries significant social weight. It influences not only how individuals are judged by others but also, in turn, how one chooses to interact with others socially. Those perceived to be younger in age may benefit from stereotyped attitudes favoring younger adults, whereas those perceived as older in age are more likely to be judged as less attractive and less approachable [1–4]. Research has also shown that older adults are treated differently and, for example, are likely to be spoken to in a louder, exaggerated manner, and treated as though they are weak and dependent [5, 6]. Thus, one’s age can have a notable impact on one’s social interactions.

Although perceived age can influence our social interactions in various ways, the perceptual judgment of age is a subjective decision process and can be easily influenced by various age-irrelevant variables, often resulting in an incorrect judgment (e.g., misjudging biological age). For example, the perceiver’s age has been shown to relate to more accurate judgments of those of a similar age compared to those not as close in age, although frequent contact with other age groups can minimize this difference [7]. Because age judgments are often first made from an assessment of the face, it is further important to recognize that facial characteristics such as the quantity and depth of wrinkles, the skin texture, and the facial shape can cause an individual to be judged as older than their actual age [8, 9].

Whether we smile or frown, it never changes our biological age. However, emotional expression may be a significant determinant of perceived age [7]. This is thought to be due, in part, to age stereotypes that tend to look unfavorably on older age. For example, younger individuals tend to be judged as more attractive, likable, and having more energy than older adults [2, 10]. Thus, a positive facial expression could be expected to trigger positive stereotypes when assessing one’s age, and negative facial expressions might have the opposite effect. Indeed, smiling makes people appear friendlier and more attractive than displaying a neutral expression [11], which parallels how younger adults are perceived more positively than older. Smiling also has been found to be associated with appearing to have a babyish face, which also impacts judgments towards a younger age [12]. Although intuition from experience and evidence from aging stereotype literature tells us that a positive facial expression makes us look younger than we really are and negative facial expression makes us look older, surprisingly little empirical evidence exists to explain how age-irrelevant emotional expressions bias subjective age perception. A recent study that investigated the accuracy of age estimation showed that the age of a face with a neutral expression was more accurately estimated compared to other expressions, while the age of a face showing a happy facial expression was underestimated compared to its actual age [7]. However, this study employed only three age groups of faces, with faces clumped into young, middle-aged, and old groups. Thus, except age estimation accuracy information for very specific ranges of faces, they could not explain how the *perceptual decision threshold* of young versus old categorization (i.e., the age level at which perceptual decision shifts from young to old categorization) would be modulated by the emotional expression of a face in a *continuum* of age. That is to say, the Voelkle et al. (2012) study could not explain why middle-aged people (e.g., 40s and 50s) can be perceived as either young or old based on their emotional expression. Given that age perception in social interaction usually occurs along a continuum of age rather than at the extremes, it is important to understand the systematic effect of the emotional expression on the decision threshold of age perception, which has not been investigated yet.

The present study aims to explore whether task-irrelevant facial emotion can influence decisions about age (i.e., young vs. old categorization). We hypothesize that the emotional expression of a face will systematically bias judgments of its age. Specifically, we predict that faces with sad expressions will be judged as old sooner (e.g., at younger ages) than faces with neutral expressions. Similarly, we expect happy faces to be categorized as young later (e.g., into higher ages) than neutral faces. In other words, sad faces are expected to decrease the age at which a face is considered old based on age-related stereotype attitudes associating negative emotions with older age [13]. In a similar way, happy faces are expected to increase this perceptual threshold compared to neutral faces and show a bias towards being perceived as younger due to their positive expressivity as has been conceptually shown in other research [7, 12]. We also hypothesize that perceptual judgment shifts by emotional expressions would be accompanied by increased reaction times for young and old categorization that reflect additional cognitive resource deployment for emotional decoding in the age-decision process. Specifically, we anticipate that it will take participants longer to judge stereotype-inconsistent faces (i.e., young, sad faces and old, happy faces) because participants will need more cognitive resources to process the inconsistent information and execute their age judgment.

## Materials and Methods

### Participants

Participants included thirty-eight healthy college students with a mean age of 21.37 years (*SD* = 4.25; 27 women) who signed up through the Psych Pool online research participant recruitment system at the University of Missouri—Kansas City. Target sample size (40 subjects) was determined based on our previous studies [14–17], and data collection was stopped by the end of the academic semester. One additional subject participated but was excluded due to unreliable chance-level responses. Participants received course credits for completing the experiment. The study protocol was approved by Institutional Review Board of University of Missouri—Kansas City (IRB 13–795). All participants provided their written informed consent prior to study participation following the approved study procedure.

### Experimental Task

**Fig 1:**
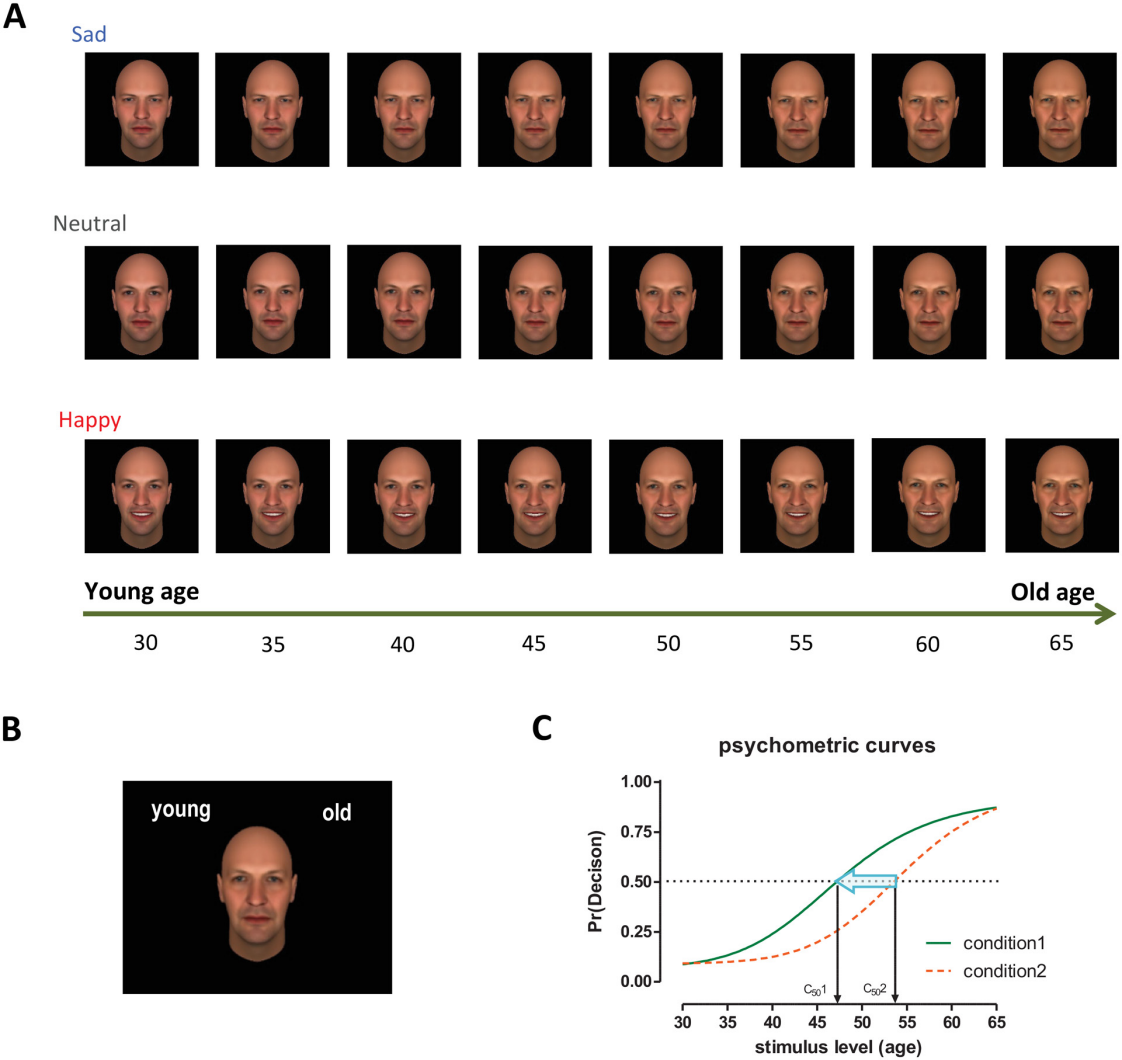
**A. Experimental stimuli used for the age judgment task.** All facial stimuli were computer-generated and no actual faces were used in our study. The emotional expression (sad, neutral, or happy) and age of the facial stimulus were manipulated by using morphing software. Faces of all emotional expressions have eight equivalent age gradients ranging from 30 years old to 65 years old increasing by 5-year increments. **B. Sample screen of the young-old judgment task.** Participants were asked to make an age decision in two-alternative forced-choice (either young or old) procedures. The positions of young and old labels were counterbalanced across participants. **C. Psychometric curves (Naka-Rushton contrast response model).** X-axis represents stimulus intensity level and Y-axis represents response probability. In our experiments, the stimulus intensity represents the incremental increase of age of morphed faces (30 to 65 years old) and the response represents the proportion of old decisions in a forced two-alternative choice task. The *C*_*50*_ or *PSE* (Point of Subjective Equality) parameter indicates the perceptual decision threshold. A leftward shift of the psychometric curve (see arrow) would constitute evidence for a decreased perceptual threshold for condition 1 compared to condition 2.

A novel computerized task was developed to test our hypotheses. Facial stimuli consisted of one computerized male face of ambiguous ethnicity expressing one of three emotional conditions: neutral, sad, or happy (Fig 1A). Our study used only male faces to prevent a potential confounding effect of cosmetic makeup that might occur when people judge female’s age. Each emotional condition had eight age levels. The youngest level corresponded to age 30 and the oldest level corresponded to age 65. All age levels were separated by 5 years. Facial stimuli of varying age and emotion levels were constructed using FaceGen Modeller (Singular Inversions, Toronto, Canada), which has been validated in previous literature [18–20]. The FaceGen’s face model is based on a 3D laser-scanned face database, and it parametrically adjusts faces on multiple dimensions including emotional expression and age. Age was manipulated in our task independent of emotional manipulation. Experimental control over age manipulation by using one computer-generated facial stimulus allowed us to eliminate any confounding effects that variations in physical aging processes might have across different individuals’ faces. That is to say, we were able to control the rate at which our stimulus aged to be consistent between age points, and remove any risk that photographed images might present if a face was to age drastically and quicker than other cohort members during a certain stage of life. FaceGen’s age manipulation algorithms include flattening the nose, loosening the skin around the face structure, and sagging the skin, particularly surrounding facial features. We chose to use ages ranging from 30–65 in order to center our range on ambiguous middle ages that are often not as clearly defined as “young” or “old.” Faces were created by balancing different ethnicities (African, European, East Asian, and South Asian) into our one, primary identity. Because the software requires one ethnicity to be slightly more dominant, we chose for the main task to use a European-based mixed ethnicity identity. The identities in the practice test were African- and South Asian-based (S1A Fig).

To help participants acclimate to our experimental paradigm and to provide evidence to support the general applicability of our findings, a short practice session (120 trials) with facial identities that were ethnically different from the face of the main task was performed first. The same procedures as in the main task were used, except that only 4 age levels were used (i.e., 35, 45, 55, and 65 years old) and 2 identities were shown (African and South Asian-based faces).

In the main age judgment task, participants were asked to decide whether they would categorize a facial image shown on the screen as “Young” or “Old” by pressing the keyboard key that corresponded to the respective category (Fig 1B). Participants were told to sort the facial stimuli as quickly and as accurately as possible. The schedule of stimuli presentation and data acquisition were programmed using SuperLab (Cedrus, San Pedro, CA). A white fixation-cross centered on a black screen appeared first to indicate the location of the stimuli. The top left and right corners of the screen displayed either the category “Old” or “Young” in white, 36-point font, with the specific category location (left or right) counterbalanced between participants. A face (400 by 400 pixels), centered on the screen, was presented for 100 ms after the fixation cross. The brief stimulus presentation was employed to eliminate or minimize the occurrence of deliberate eye saccades [21], as similarly done in previous studies [14–16, 22, 23]. Also, it has been shown that people can make rapid social judgments even from only brief (e.g., 100 ms) exposures of faces without significantly increasing reliability when given more time [24]. The participant sorted this face by pressing either “e” or “i” on the keyboard for the left or right category, respectively. After responding, a yellow fixation-cross (duration 500 ms) signified that responses were registered. If the participant failed to categorize a face within two seconds, the word “MISS” appeared for 500 ms. A randomized inter-trial-interval of one to two seconds displayed a blank screen with the fixation-cross before the next trial began. The task was broken into four blocks, each containing the eight age-level variations presented randomly in the happy, neutral, or sad emotional states, repeated ten times per block for a total of 240 presentations per block. The order of images across ages and emotions was random within each block for each participant in order to minimize any order or repetition effects. Each block took approximately 15 minutes, making the entire task last slightly over one hour. We planned a 3 x 8 (emotion by age) within-subjects design, and our task was constructed to allow us to observe 40 decisions (10 per block across four blocks) for each condition of interest in a total of 960 trials (i.e., 3 emotions x 8 age levels x 10 trials per block x 4 blocks).

### Psychometric Curve Fitting

We hypothesized that the emotional expressions of facial stimuli would influence age judgment on morphed faces by systematically shifting the shape of the psychometric functions. For each individual, we parameterized psychometric functions and then compared them across different emotional expression conditions. Relating the proportion of “old” decisions in forced choices to the age levels of the gradually morphed faces, we utilized a psychometric curve-fitting approach that has been employed in previous research [14–17, 22] by using the Naka-Rushton contrast response model [25] with an OLS (Ordinary Least Square) criterion.

$$response=\frac{R_{\text{max}}\times C^{n}}{C^{n}+C_{50}^{n}}+M$$

In the equation above, *response* represents the proportion of “old” decisions, *C* is the age levels of the faces, *C*_*50*_ is the age at which response is half-maximal (“threshold” or “point of subjective equality: PSE”), *n* is the parameter that represents the slope, *R*_*max*_ is the asymptote of the function, and *M* is the response at the lowest age. Given that the proportion of “old” decisions was used, the *R*_*max*_ parameter was constrained to be equal to or less than 1 and the *M* parameter was constrained to be equal or larger than 0. Parameter estimation was done with GraphPad Prism software (GraphPad Software, La Jolla, CA).

We hypothesized that negative and positive emotional expressions of faces would bias age judgment by changing the subjective perceptual decision threshold that represents the age level at which 50% “old” decisions occur (Fig 1C). This change is often described by the contrast gain model in perception research [26]. In our experimental context, we predicted a decreased old decision threshold (*C*_*50 sad*_) for sad faces (a leftward shift) and an increased old decision threshold (*C*_*50 happy*_) for happy faces (a rightward shift) compared to neutral faces.

## Results

Participants completed 960 two-alternative forced choices (young vs. old) for facial stimuli shown with different morphed ages and emotional expressions. The effect of task-irrelevant emotional expressions on the age perception was tested through repeated-measures ANOVAs and nonlinear psychometric curve fitting approaches. For all repeated-measures statistics, we employed Greenhouse-Geisser corrections.

**Fig 2:**
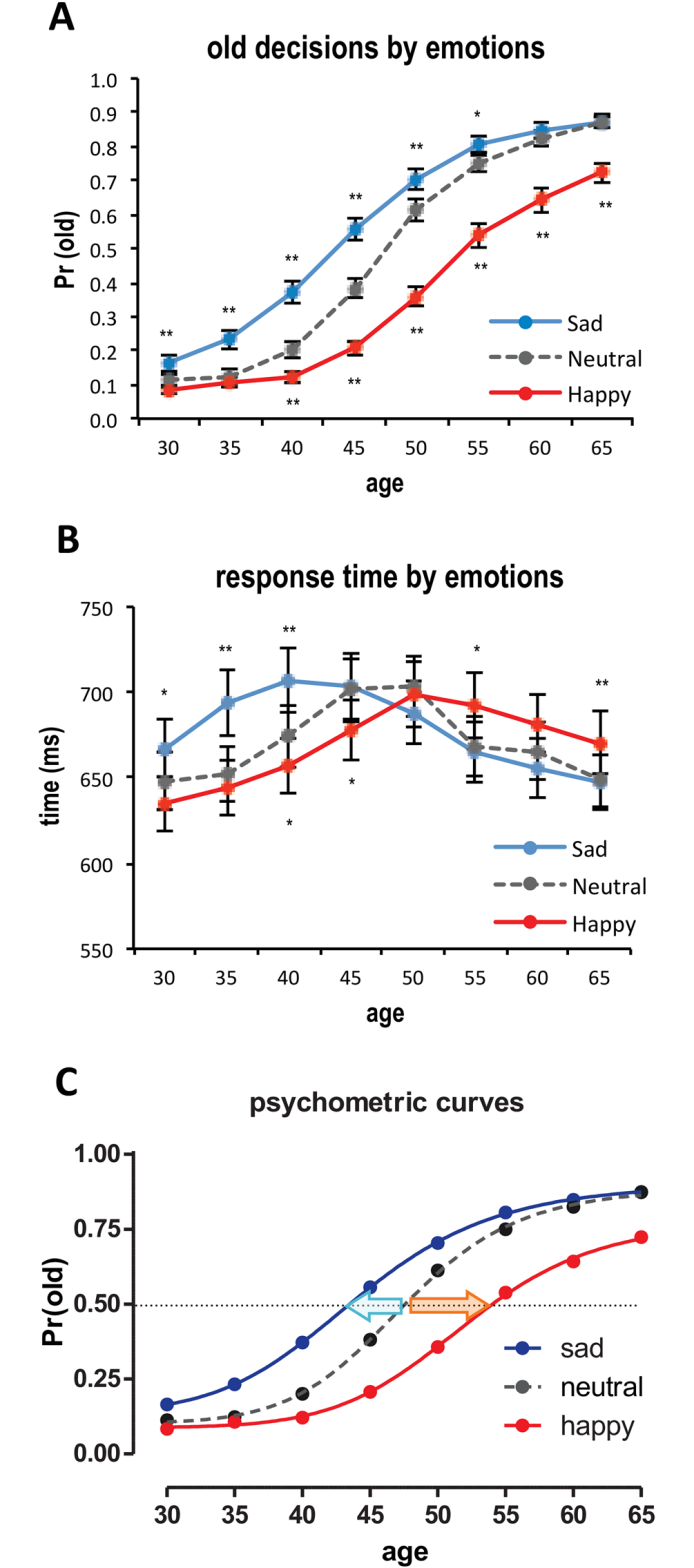
**A. Average probability of old responses as a function of age and emotional expressions of faces**. **B. Response times of age decisions.** Error bars denote the standard error of the mean. * *p* < .05, ** *p* < .01 compared to neutral faces (controls). **C. Psychometric curve fits.** For each emotional expression, psychometric curves were separately fitted by using the Naka-Rushton response function. A leftward-shift of the psychometric curve of sad faces (blue line) and a rightward-shift of the psychometric curve of happy faces (red line) compared to neutral faces (gray line) were observed. A dotted horizontal line represents the 50% probability of an old decision.

First, we performed a 3 (EMOTION: Sad, Neutral, Happy) by 8 (AGE: 30 to 65 years old in 5 years increments) repeated-measures ANOVA on the proportions of old decisions. Means and standard deviations are shown in Table 1. The ANOVA result revealed a significant 2-way interaction effect of EMOTION × AGE, *F*(14,518) = 20.66, *p* < .01, *η*_*p*_^*2*^ = .36. We also observed main effects of EMOTION, *F*(2,74) = 94.73, *p* < .01, *η*_*p*_^*2*^ = .72, and AGE, *F*(7,259) = 289.76, *p* < .01, *η*_*p*_^*2*^ = .89. To clarify the interaction effect through simple effect analyses, we performed one-way repeated-measures ANOVAs for each age levels. Then, we conducted subsequent *post-hoc* comparisons separately for sad and happy affects with neutral affect (control). For all age levels, one-way ANOVA results were significant, all *p* < .05. However, in the *post-hoc* comparisons (see Fig 2A), sad faces were more frequently judged as old compared to neutral faces in a range of the lowest age of 30 through 55 years old, 30: *F*(1,74) = 17.11, *p* < .01, *η*_*p*_^*2*^ = .32; 35: *F*(1,74) = 43.31, *p* < .01, *η*_*p*_^*2*^ = .54; 40: *F*(1,74) = 65.10, *p* < .01, *η*_*p*_^*2*^ = .64; 45: *F*(1,74) = 46.85, *p* < .01, *η*_*p*_^*2*^ = .56; 50: *F*(1,74) = 9.42, *p* < .01, *η*_*p*_^*2*^ = .20; 55: *F*(1,74) = 5.69, *p* < .05, *η*_*p*_^*2*^ = .13. As predicted, positive affect revealed the opposite pattern of results. Happy faces were less frequently judged as old compared to neutral from 40 to the highest age of 65 years old, 40: *F*(1,74) = 16.81, *p* < .01, *η*_*p*_^*2*^ = .64; 45: *F*(1,74) = 64.39, *p* < .01, *η*_*p*_^*2*^ = .56; 50: *F*(1,74) = 109.22, *p* < .01, *η*_*p*_^*2*^ = .75; 55: *F*(1,74) = 64.50, *p* < .01, *η*_*p*_^*2*^ = .65; 60: *F*(1,74) = 43.91, *p* < .01, *η*_*p*_^*2*^ = .54; 65: *F*(1,74) = 38.30, *p* < .01, *η*_*p*_^*2*^ = .51. These findings explain the interaction effect we observed—the effect of task-irrelevant negative and positive emotional expressions of facial stimuli interacted with the morphed age levels.

**Table 1:** Means and standard deviations of the proportion of old decisions.

Age Level of Morphed Faces								
Emotions	30	35	40	45	50	55	60	65
Sad	.165 (.159)	.232 (.169)	.372 (.198)	.556 (.202)	.704 (.184)	.806 (.165)	.848 (.148)	.874 (.114)
Neutral	.113 (.129)	.123 (.145)	.201 (.153)	.381 (.168)	.612 (.197)	.750 (.156)	.824 (.135)	.873 (.135)
Happy	.084 (.087)	.108 (.090)	.121 (.103)	.207 (.126)	.357 (.169)	.538 (.212)	.642 (.221)	.723 (.186)

Second, we performed a similar repeated-measures ANOVA on the response time data. The means and standard deviations of response times are shown in Table 2. The ANOVA result showed a significant interaction effect of EMOTION × AGE, *F*(14,518) = 8.41, *p* < .01, *η*_*p*_^*2*^ = .19. Also, main effects of EMOTION, *F*(2,74) = 3.53, *p* < .05, *η*_*p*_^*2*^ = .09, and AGE, *F*(7,259) = 8.48, *p* < .01, *η*_*p*_^*2*^ = .19, were significant. Again, we performed simple effect analyses for each age levels. Except for the 50-year-old level, all other one-way ANOVA results were significant, all *p* < .05. As shown in Fig 2B, at the low levels of age (30, 35 and 40 years old), decision times for sad faces were significantly longer than neutral faces, 30: *F*(1,37) = 5.75, *p* < .05, *η*_*p*_^*2*^ = .13; 35: *F*(1,37) = 26.69, *p* < .01, *η*_*p*_^*2*^ = .42; 40: *F*(1,37) = 14.27, *p* < .01, *η*_*p*_^*2*^ = .28. Interestingly, decision times for happy faces were significantly longer than neutral faces at the high levels of age (55 and 65 years old), 55: *F*(1,37) = 5.59, *p* < .05, *η*_*p*_^*2*^ = .13; 65: *F*(1,37) = 7.50, *p* < .01, *η*_*p*_^*2*^ = .17. Combined with the decision data above, the response time results imply that the increase of old decisions for sad faces at the low age levels and the decrease of old decisions for happy faces at the high age levels were related to additional cognitive processes (and thus, longer response times) in age judgments of emotionally charged faces compared to neutral faces.

**Table 2:** Means and standard deviations of response times in millisecond.

Age Level of Morphed Faces								
Emotions	30	35	40	45	50	55	60	65
Sad	667 (106)	693 (118)	707 (114)	703 (118)	688 (112)	665 (109)	656 (106)	647 (99)
Neutral	648 (105)	652 (101)	674 (111)	702 (115)	703 (108)	669 (108)	666 (104)	648 (97)
Happy	635 (100)	644 (98)	657 (100)	678 (106)	699 (116)	692 (115)	682 (103)	670 (111)

As described earlier, we postulated that the emotional expressions of facial stimuli would systematically bias age judgment. More specifically, we hypothesized that the perceptual decision thresholds that determine binary categorical decisions (young vs. old) would be differentially modulated by the task-irrelevant affect of facial stimuli, resulting in more frequent old decisions for sad faces (i.e., a decrease of old decision threshold by the presence of negative affect) and less frequent old decisions for happy faces (i.e., an increase of old decision threshold by the presence of positive affect) compared to neutral faces. The decision threshold changes were tested by comparing the psychometric curve fit parameters. In the Naka-Rushton contrast response model, *C*_*50*_ parameter represents the decision threshold. The means of *C*_*50*_ parameters for sad, neutral, and happy faces were 44.25 (*SD* = 5.49), 47.94 (*SD* = 4.91), and 53.32 (*SD* = 6.68), respectively. A repeated-measures ANOVA on *C*_*50*_ parameters showed a significant effect of EMOTION, *F*(2,74) = 30.23, *p* < .01, *η*_*p*_^*2*^ = .45. In simple effect analyses, as expected, we found a significant decrease in the old decision threshold in sad faces, *F*(2,74) = 8.79, *p* < .01, *η*_*p*_^*2*^ = .19, as well as a significant increase in the old decision threshold in happy faces compared to neutral faces, *F*(2,74) = 28.38, *p* < .01, *η*_*p*_^*2*^ = .43. These findings imply a leftward shift of the psychometric curve in sad faces and a rightward shift of the psychometric curve in happy faces (Fig 2C). In other words, experimental face stimuli with neutral expressions were more likely judged as old when their morphed ages were above 47.94 years old. However, sad faces were more likely judged as old when ages were above 44.25 years old, and, interestingly, happy faces were more likely judged as old when ages were above 53.32 years old. Note that although these three ages may socially be labeled as “middle aged” instead of old in everyday life, the combination of the binary categorization and the restricted age range of faces from 30–65 forced participants to divide the faces into two categories (young vs. old), which allowed us to systematically detect subjective perceptual decision threshold. The average variability by emotional expressions (positive − negative) across participants was 9.07 years (95% *CI*: 5.94 ~ 12.20). For completeness, we performed similar exploratory analyses on other parameters of psychometric curve fits (*M*, *R*_*max*_, and *n*) although there was no specific hypothesis about these parameters (Table 3). All other psychometric curve parameters of sad and happy faces were not significantly different from neutral faces, all *p* > 05.

**Table 3:** Means and standard deviations of psychometric curve fit parameters.

Emotions	*C*_*50*_	*R*_*max*_	*n*	*M*
Sad	44.25 (5.49)	.78 (.22)	16.05 (7.78)	.14 (.15)
Neutral	47.94 (4.91)	.81 (.16)	13.28 (4.62)	.09 (.11)
Happy	53.32 (6.68)	.76 (.23)	17.93 (20.55)	.08 (.08)

## Discussion

Our study supports social intuition that a smile makes us look younger while a frown makes us look older with novel empirical evidence from a young adult sample. Examining the process of age judgment not only has important value in better understanding the psychological mechanisms that influence our perception but also has significant implications in U.S. society, as the number of adults living longer increases dramatically. The present study investigated the role that emotions play in biasing the categorization of a face as young or old when age ranged on a continuum. Using the two-alternative, forced choice task that we designed for this study allowed us to better determine the threshold shift in age judgments and more clearly illuminate the role that emotion played in these judgments.

Our finding that sad faces were more often judged as old compared to neutral faces supports our first hypothesis. Sad faces were more often labeled as old at the younger years (ages 30 ~ 55) than neutral faces—a leftward shift in the psychometric function (i.e., favoring old judgments). In support of our second hypothesis, we also found that positive affect, specifically happiness, biased age judgments such that happy faces were less often labeled as old compared to neutral at the upper age levels (ages 40 ~ 65)—a rightward-shift in the psychometric function (i.e., favoring younger judgments). This supports the findings of previous research that have found an underestimation of age when a happy emotion was expressed [7, 12], not an overestimation of age [27]. To note, the same ability of emotional expression to bias middle-range, ambiguous age judgments was also found during practice test trials which used African and South Asian facial identities (S1B Fig), suggesting that our findings were not limited to the specific facial stimuli used in the main task. However, given the brevity of the practice task and our sample (i.e., U.S. college students), it is somewhat difficult to confidently draw conclusions across ethnicities or cultures, which should be answered in future studies.

Contrary to our findings of an underestimation of age by positive expressions, one study found that smiling faces were perceived as older (based on the average age estimated pooled across all facial stimuli presented) in photographed images, potentially due to the exaggeration of wrinkles when smiling as compared to when neutral [27]. However, the photographs (smiling and neutral facial images from a face photo database) in this study only ranged from 20–40 years old and did not control for rates of aging across individuals, as we were able to in our study by employing computer generated faces. Therefore, this counterintuitive finding may reflect an idiosyncratic effect of smile-associated wrinkles in younger adults rather than representing a general effect of positive emotional expressions on age perception in a continuum of aging process from younger adulthood to older adulthood of one identical individual. On the other hand, Wang and colleagues (2015) also used photographed images and found that how babyish a face looked mediated the relationship between a smiling expression and age underestimation [12]. However, our study, using computer-generated, systematically manipulated images, was able to eliminate any potential variations in babyish features and aging rates of individual identities and thus provides a new perspective from previous research.

We further found evidence to support our hypothesis concerning the role that emotion would play in influencing the reaction times at different levels of age judgments. Judgment times for sad faces at the youngest levels of age range (ages 30 ~ 40) were significantly longer compared to neutral faces. Conversely, decision times on happy faces were significantly longer at high age levels (ages 55 and 65). These findings would suggest that additional processing resources were needed to take into account the emotional expression differences between these conditions. Intuitively, one might expect that age decisions at the highest and lowest age levels would be the easiest to decipher and would require the least amount of cognitive processing, thus being the quickest. The overlap between the significantly longer response times and the significantly different age judgments for both emotions can be explained by the idea that emotional expression appears to be playing a moderating role in age judgments. Decisions incorporating both that the face is young and sad, or reversely, that it is old and happy, would require additional effortful processing of information that is counter to aging stereotypes.

Our findings suggest that emotion played a particularly strong role in age judgment within the middle range of ages. The middle ages were most critical in that the perceptual switch between young and old age judgments occurred in this range, however they were also the most ambiguous to judge for this same reason. It would appear that the ambiguity in attempting to judge a face from clues about its age forced our young-adult participants to dig deeper and use irrelevant cues, i.e. emotional expressions, in order to aid in the required judgment process.

Then, why do happy expressions make people look younger while sad expressions make people look older? Based on aging stereotypes research [2, 4], we speculated that the favorable association between positive emotions and younger ages and unfavorable association between negative emotions and older ages would produce an increase of old decision threshold (i.e., underestimation of age) by positive expressions and a decrease of old decision threshold (i.e., overestimation of age) by negative expressions. The stereotypical and non-stereotypical appearance (e.g., old = sad and young = happy) of facial stimuli nicely explains our results. However, this would mean that it is not the emotion per se but the stereotypical attitude we have about young and old age groups that is an important determinant or mediator of our findings—a topic that would be very interesting to pursue for future studies. Whereas this stereotypical attitude-related speculation is in accordance with aging stereotypes research, there are other possibilities we can consider to interpret underlying psychological mechanisms that explain our findings. For example, it has been known that happy faces have social advantages that increase prosocial motivation [28] as well as approach motivation [29]. Indeed, a happy expression is a universal sign that represents positive prosocial intention, which has been demonstrated in even most remote cultures [30]. This prosocial and approach motivation that a happy expression conveys, in turn, might lead the participants to judge happy facial stimuli as being younger based on the previous findings that younger people are perceived as more approachable than older people [2, 4]. Another explanation would be the easier processing (i.e., more rapid and efficient cognitive processing) of happy faces compared to sad faces [31]. Happy expressions are known to be more easily discriminable than other emotional expressions [32]. Thus, an easier processing or detection advantage in the face-processing stream might lead to more favorable (= younger) judgments in our experiment. Still another plausible explanation is that happy faces might induce a happy mood and sad faces might induce a sad mood in a perceiver that, in turn, might let a participant judge happy faces more favorably (= younger) and judge sad face less favorably (= older) in a mood-congruent way. Also, a recent study shows that mood induction can affect local facial feature discrimination performance (e.g., participants induced to be happy better detected changes in eyes than participants induced to be sad) [33]. Thus, induced mood and its subsequent effect on face processing in a perceiver might mediate the effect of facial expression on age judgment. Taking these alternatives together, further elucidation of the underlying psychological mechanisms would be a highly relevant and interesting topic for future studies.

Certain limitations should be noted when interpreting the findings of our study. First, the images were generated by using computer software. While this allowed us to minimize individual facial differences in aging and expressions and increase the internal validity, it sacrificed some of the possible generalization of our findings. However, given that previous research [9, 34] has successfully implemented similar methods, we do not consider our image choice to be a major limitation. Indeed, having the ability to systematically manipulate age allowed us to precisely control for facial variations such as differences in wrinkles and skin spots that may have arisen from natural, photographed images. Further, we were able to control a potential ethnicity effect by manipulating a face stimulus across multiple ethnicities. To our knowledge there exists no stimulus database of the same facial identity photographed across multiple decades with differing facial expressions that would have suited our research. It is also important to note that our methods only explored the affect of sadness and happiness on age judgments, and thus the interpretation of the results is limited to these emotions. However, it might be plausible that other negative expressions such as anger, disgust, or fear have similar effects on age perception, if they share similar stereotypical associations or psychological mechanisms. Further research should be done to determine the impact of different types of emotions. The sample we used consisted of relatively young adults. Our intent was to focus on the age categorization of stimuli and not on the age of the judges; however, it is important to address concerns that may arise from not intentionally recruiting older adults. If the “own-age” advantage of a relatively young adult sample was indeed a confound [8], we might expect that all younger faces would be more accurately categorized compared to old, regardless of expressed emotion. Still, it remains important to note that our findings are only generalizable to a young adult population and that it is still possible that older adults may perform differently. The stimuli we utilized were only male faces for the purpose of experimental control (i.e., a potential confounding effect of cosmetic makeup of female faces), which is a limitation in that the results cannot be generalized to how female faces (with and without cosmetics) might be judged. Finally, our participants were predominantly female, which may also be seen as a potential limitation. But, we could not observe any significant *C*_*50*_ parameter difference between male and female participants, all *p* > .05. However, when considering the relatively small size of this study, null findings should be interpreted cautiously. Furthermore, research has shown that female perceivers are more accurate than males in assessing age [35], and, additionally, that male stimuli are more accuracy judged for age than female stimuli [36]. These findings would work against our aim and, if true, make our research hypothesis more difficult to prove based on the methods we used. If females are more accurate as perceivers, and male faces are more accurately judged on age than female, then it might be expected that both of these factors would make the age judgment more robust and less susceptible to the influence of our emotional manipulation. Given that this study was exploratory, it is worthwhile to highlight the importance that finding age judgment biases, even in a limited sample (only young adults), means in terms of our conceptualization of perceptual judgments. However, to augment these findings, future research that implements a more diverse sample, set of stimuli, or expressed emotions may be useful in determining the overall generalizability of the results.

This study is one of the first of its kind to provide scientific evidence of the role emotion plays in the process of categorizing a face’s age, a process in which emotional expression would initially appear to be unrelated. Going beyond previous research [7, 12], we were able to show that emotional expression systematically impacted age estimates by young adults in a way consistent with age bias research. Specifically, the pro-young age bias shown in age stereotype research [2, 37] was congruent with our findings that happy expressions made a face look younger and sad expressions made a face look older than neutral. Our results would suggest a true advantage in displaying a happy facial expression over a neutral expression when attempting to be regarded as young, and a strong disadvantage in bearing a sad facial expression. Given research on negative aging stereotypes, and the social disadvantages that can occur when judged to be older [3], it is critical to note the results of this study and how emotion is capable of modulating the perceived age of a face.

## Supporting Information

S1 Fig **A. Experimental stimuli used for the practice task.** Two different identities were used for the practice task. Faces of all three emotional expressions have eight equivalent age gradients ranging from 35 years old to 65 years old increasing by 10-year increments. **B. Average probability of old responses as a function of age and emotional expressions of faces**. *N* = 28 (Due to a technical issue, practice task responses were not recorded for first 10 subjects). Error bars denote the standard error of the mean. * *p* < .05, ** *p* < .01 compared to neutral faces (controls).
(TIF)
