# Supplementary figures and images for: Be Happy Not Sad for Your Youth: The Effect of Emotional Expression on Age Perception
Source: PLoS One. 2016 Mar 30;11(3):e0152093. doi: 10.1371/journal.pone.0152093 (PMC4814130; doi:10.1371/journal.pone.0152093)

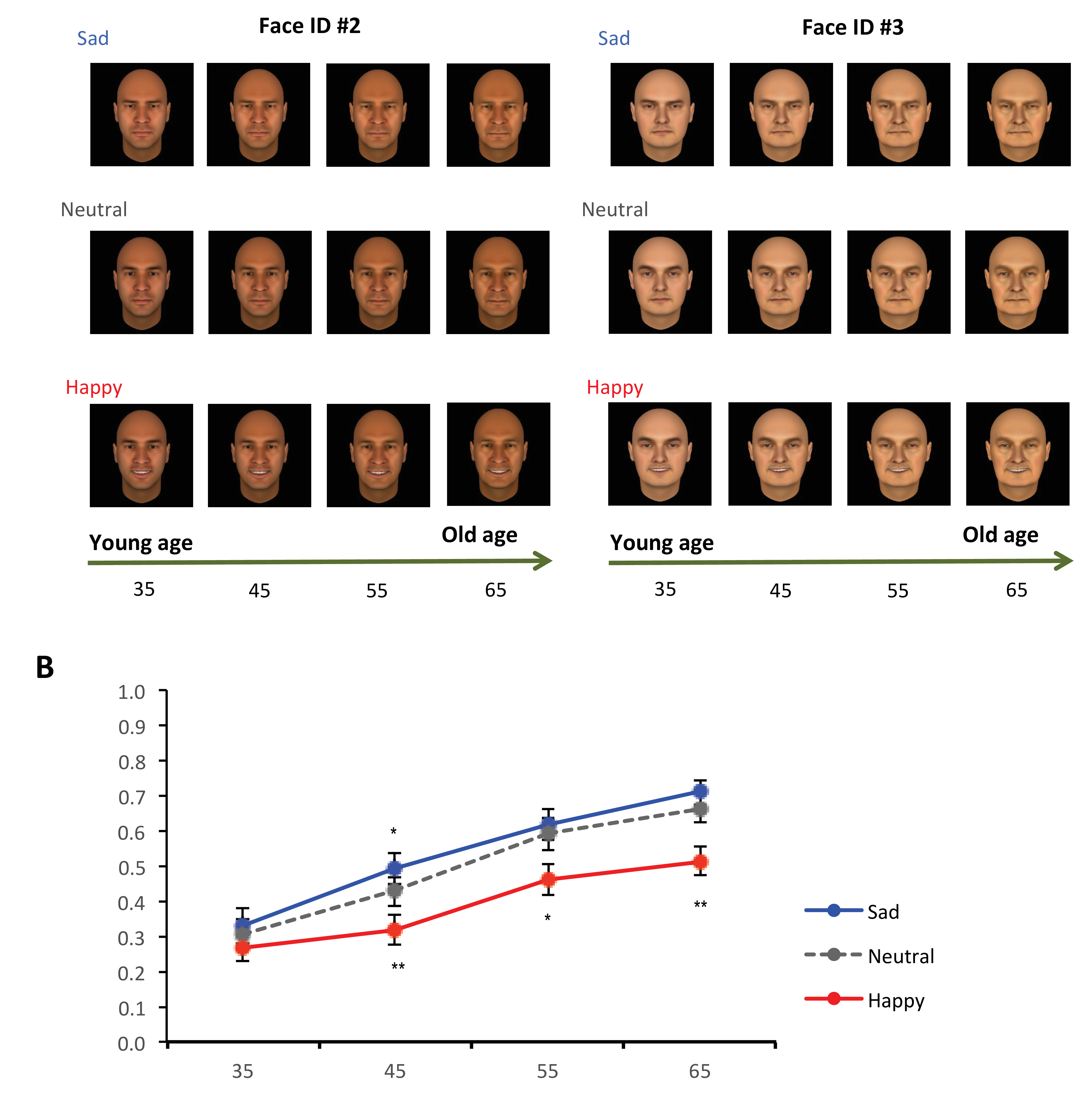

Supplement: S1 Fig — A. Experimental stimuli used for the practice task. Two different identities were used for the practice task. Faces of all three emotional expressions have eight equivalent age gradients ranging from 35 years old to 65 years old increasing by 10-year increments. B. Average probability of old responses as a function of age and emotional expressions of faces. N = 28 (Due to a technical issue, practice task responses were not recorded for first 10 subjects). Error bars denote the standard error of the mean. * p < .05, ** p < .01 compared to neutral faces (controls). (TIF) [file pone.0152093.s001.tif]
